# Transportin-3 Facilitates Uncoating of Influenza A Virus

**DOI:** 10.3390/ijms23084128

**Published:** 2022-04-08

**Authors:** Jiahui Zou, Luyao Yu, Yinxing Zhu, Shuaike Yang, Jiachang Zhao, Yaxin Zhao, Meijun Jiang, Shengsong Xie, Hailong Liu, Changzhi Zhao, Hongbo Zhou

**Affiliations:** 1 State Key Laboratory of Agricultural Microbiology, College of Veterinary Medicine, Huazhong Agricultural University, Wuhan 430070, China; zoujiahui@webmail.hzau.edu.cn (J.Z.); luyaoyu0408@163.com (L.Y.); yingzizhu@163.com (Y.Z.); yangshuaike0910@163.com (S.Y.); jiachangzhao1223@163.com (J.Z.); YaxinZhao@webmail.hzau.edu.cn (Y.Z.); jiangmeijun1996@163.com (M.J.); 2 Key Laboratory of Preventive Veterinary Medicine in Hubei Province, The Cooperative Innovation Center for Sustainable Pig Production, Wuhan 430070, China; 3 Key Laboratory of Agricultural Animal Genetics, Breeding and Reproduction of Ministry of Education & Key Lab of Swine Genetics and Breeding of Ministry of Agriculture and Rural Affairs, Huazhong Agricultural University, Wuhan 430070, China; ssxie@mail.hzau.edu.cn (S.X.); hailongliu@webmail.hzau.edu.cn (H.L.); czzhao@webmail.hzau.edu.cn (C.Z.)

**Keywords:** TNPO3, CRISPR/Cas9, influenza virus, uncoating

## Abstract

Influenza A viruses (IAVs) are a major global health threat and in the future, may cause the next pandemic. Although studies have partly uncovered the molecular mechanism of IAV–host interaction, it requires further research. In this study, we explored the roles of transportin-3 (TNPO3) in IAV infection. We found that TNPO3-deficient cells inhibited infection with four different IAV strains, whereas restoration of TNPO3 expression in knockout (KO) cells restored IAV infection. TNPO3 overexpression in wild-type (WT) cells promoted IAV infection, suggesting that TNPO3 is involved in the IAV replication. Furthermore, we found that TNPO3 depletion restrained the uncoating in the IAV life cycle, thereby inhibiting the process of viral ribonucleoprotein (vRNP) entry into the nucleus. However, KO of TNPO3 did not affect the virus attachment, endocytosis, or endosomal acidification processes. Subsequently, we found that TNPO3 can colocalize and interact with viral proteins M1 and M2. Taken together, the depletion of TNPO3 inhibits IAV uncoating, thereby inhibiting IAV replication. Our study provides new insights and potential therapeutic targets for unraveling the mechanism of IAV replication and treating influenza disease.

## 1. Introduction

Influenza A virus (IAV) is a negative-stranded and segmented RNA virus belonging to the Orthomyxoviridae family [1]. IAV infects humans and animals in the upper respiratory and causes seasonal epidemics and occasional pandemics, posing an enormous threat to the global economy and public health [2,3,4]. As IAV has the ability to rapidly evolve to generate new reassortment viruses, conventional antiviral therapies including vaccines, neuraminidase inhibitors, and M2 channel blockers have limited antiviral efficiency [5,6]. The development of new antiviral therapeutics against IAV is urgently needed.

IAV relies on a host’s machinery to replicate and accomplish its life cycle [7]. The IAV–host interactions have been partially revealed [8,9,10,11,12,13], but they still require further research. The virus’ evolution of resistance to host-targeted therapeutics is deemed to be slow, thus the development of host-directed therapeutics against IAV is imperative. The early life cycle of IAV can be divided into six steps: adsorption, endocytosis, acidification, endosomal transport, uncoating, and the import of viral ribonucleoprotein (vRNP) into the nucleus [14,15]. IAV hemagglutinin (HA) combines with the sialic acid receptor on the surface of the host cell, then virus particles are internalized by clathrin-mediated endocytosis, micropinocytosis, clathrin- and caveolin-independent endocytosis, or caveolin-mediated endocytosis [16,17,18]. Subsequently, the early endosome is formed and moved toward the nucleus along cytoskeleton proteins. When the virus particles are exposed to the low pH of 5.0, HA undergoes a conformational change [19], inducing the fusion of the viral particle membrane and the late endosomal membrane [13,20,21]. The capsid is released into the cytoplasm, and matrix protein M1 and vRNPs are separated from each other. Subsequently, vRNPs are imported into the nucleus for transcription and replication [22,23]. Any interference or interruption in these steps will interrupt the IAV replication. Host factors screened to be required for IAV replication can act as host-targets utilized to develop therapeutics against IAV.

TNPO3, a member of the importin β superfamily, participates in regulating nucleo-cytoplasmic translocation of importins, exportins, and their specific binding cargos [24]. The process is regulated by the small GTPase Ran, which is predominantly in a GDP- and GTP-bound form [25]. TNPO3 specifically mediates the nuclear import of splicing factor serine/arginine (SR) proteins, such as RBM4, SFRS1, SFRS2, and CPSF6 [26,27,28,29]. Cyto-plasm cargos bound with importins competitively bind to RanGTP, inducing release into the nucleus [30]. Recently, several studies have shown that TNPO3 is implicated in the replication of several viruses. TNPO3 regulates human immunodeficiency virus-1 (HIV-1) replication through interacting with HIV-1 integrase and capsid proteins [31,32,33]. Knockout of TNPO3 also restrained the import of prototype foamy virus (PFV) integrase and induced unintegrated DNA in PFV-infected cells [34,35]. However, the roles of TNPO3 in IAV replication have not been reported. Transportin-1 (TNPO1), a homologous protein of TNPO3 that binds to a PY-NLS sequence motif close to the amino terminus of the matrix protein M1, promotes the removal of M1 and induces disassembly of vRNP bundles [23]. We constructed the TNPO3 knockout newborn pig trachea epithelial (NPTr) cells by CRISPR/Cas9 technology [36]. TNPO3 was validated to be involved in IAV uncoating and the subsequent vRNP importin steps, but was not necessary for IAV attachment, endocytosis, and endosomal acidification. For analysis of the crucial roles that viral M1 and M2 proteins perform in IAV uncoating [22,37] and the PY-NLS-like motifs, which were found to be located in viral M1 and M2 [23,38], we also verified the interaction and colocalization between TNPO3 and the viral M1 and M2 proteins. This interaction may be the reason behind TNPO3’s regulation of IAV uncoating.

Taken together, we validated the critical role of TNPO3 in IAV infection. Stepwise dissection of the viral entry process verified that TNPO3 knockout restrained the IAV uncoating and the subsequent vRNP’s importin steps, which may take place through the interactions between TNPO3 and the viral M1 and M2 proteins. This study provides new insight into the IAV replication mechanism and a potential host-directed therapeutics target.

## 2. Results

### 2.1. TNPO3 Is Involved in the IAV Replication

**Figure 1:**
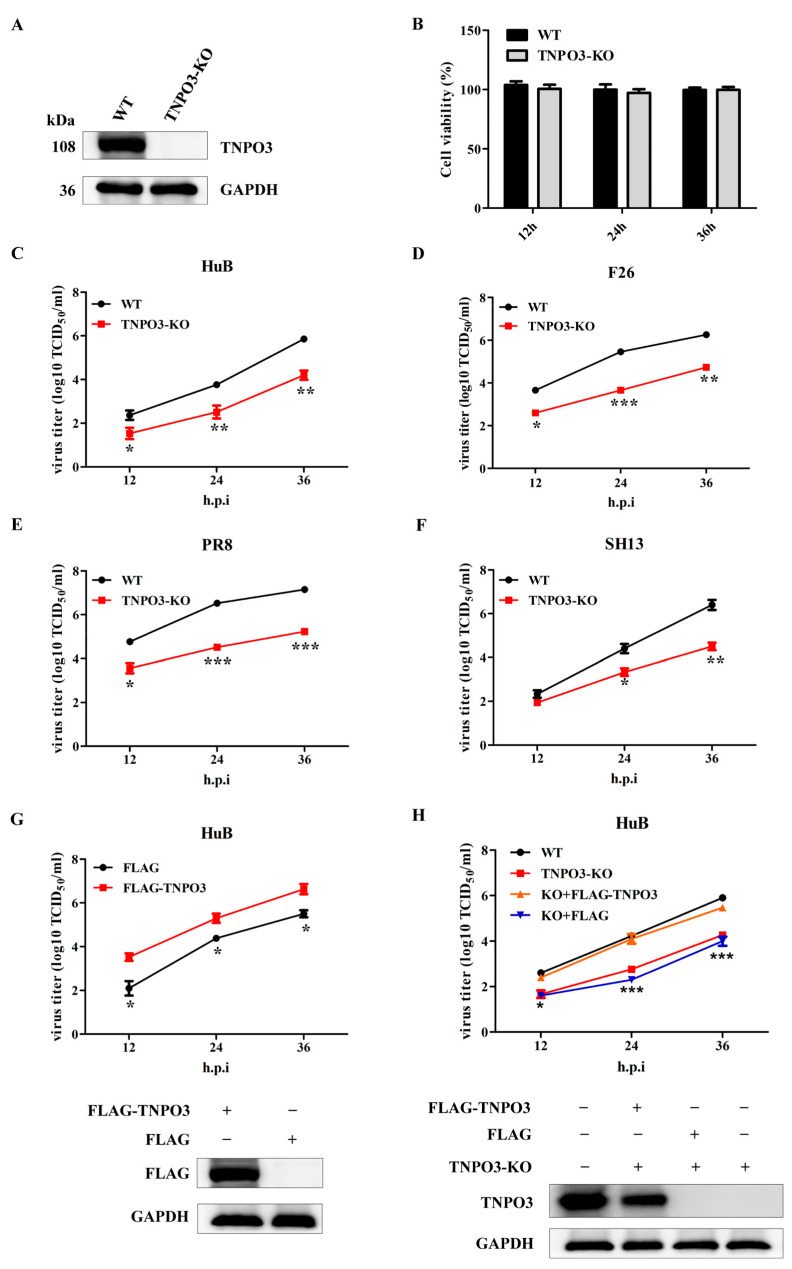
TNPO3 is involved in the replication of IAV. (**A**) Western blot detection of the TNPO3 in knockout (KO) and wild-type (WT) cells. (**B**) Cell viability was determined using CCK-8 detection. (**C**–**F**) Assessing the effects of TNPO3 knockout on IAV replication with the TCID_50_ assay. WT and TNPO3-KO cells were infected with HuB/H1N1, F26/H1N1, PR8/H1N1, and SH13/H9N2 at MOI of 0.01, respectively. (**G**) Assessment of the effect of overexpression of TNPO3 on HuB/H1N1 virus (MOI = 0.01) replication by the TCID_50_ assay. (**H**) TNPO3 knockout cells were complemented with wild-type TNPO3, and then challenged with HuB/H1N1 virus (MOI = 0.01). FLAG-TNPO3—overexpression vector of 3 × FLAG-CMV-TNPO3. FLAG—control vector of FLAG. Virus titers were determined by TCID_50_ assay on MDCK-NBL-2 cells (mean ± SD of three independent experiments; *, *p* < 0.05; **, *p* < 0.01; ***, *p* < 0.001; two-tailed Student’s *t*-test).

To study the function of TNPO3 in IAV infection, we generated TNPO3 knockout (TNPO3-KO) NPTr cells with CRISPR/Cas9 technology. The knockout effect of TNPO3 was determined by Western blot, and the results revealed that the TNPO3 protein was knocked out (Figure 1A). Sanger sequencing confirmed the presence of 7 bp deletions in TNPO3-KO cells (Figure S1). Meanwhile, knockout of TNPO3 had no significant effect on the cell viability when compared to the wild type (WT) cells (Figure 1B).

Next, we explored the role of TNPO3 in the replication of IAV. TNPO3-KO cells and WT cells were infected with A/swine/Hubei/221/2016 (HuB/H1N1), and virus titers of indicated timepoints were determined. The results revealed that knockout of TNPO3 significantly inhibited the proliferation of swine influenza virus HuB/H1N1 (Figure 1C). To determine whether TNPO3 was crucial for replication in multiple influenza virus strains, the TNPO3-KO cells and WT cells were also infected with A/swine/Henan/F26/2017 (F26/H1N1), A/Puerto Rico/8-SV14/1934 (PR8/H1N1), and A/chicken/Shanghai/SC197/2013 (SH13/H9N2). The virus titers at different timepoints were also determined, suggesting that knockout of TNPO3 prominently restrained all of the aforementioned viruses’ replication (Figure 1D–F). To eliminate the interference of other factors, the complement of exogenous TNPO3 cDNA in WT cells (Figure 1G) or TNPO3 KO cells (Figure 1H) significantly increased the proliferation of HuB/H1N1 at different timepoints post infection, indicating the participation of TNPO3 in the replication of IAV. We also examined whether TNPO3 is crucial for other viruses replication, such as the Japanese encephalitis virus (JEV) and vesicular stomatitis virus (VSV). We infected the WT and TNPO3-KO cells with JEV or VSV, and the results indicated that knockout of TNPO3 did not affect the replication of JEV and VSV (Figure S2). Together, all results demonstrated that TNPO3 was involved in IAV replication.

### 2.2. TNPO3 Is Not Required for the IAV Attachment and the Expression of Sialic Acid Receptors

**Figure 2:**
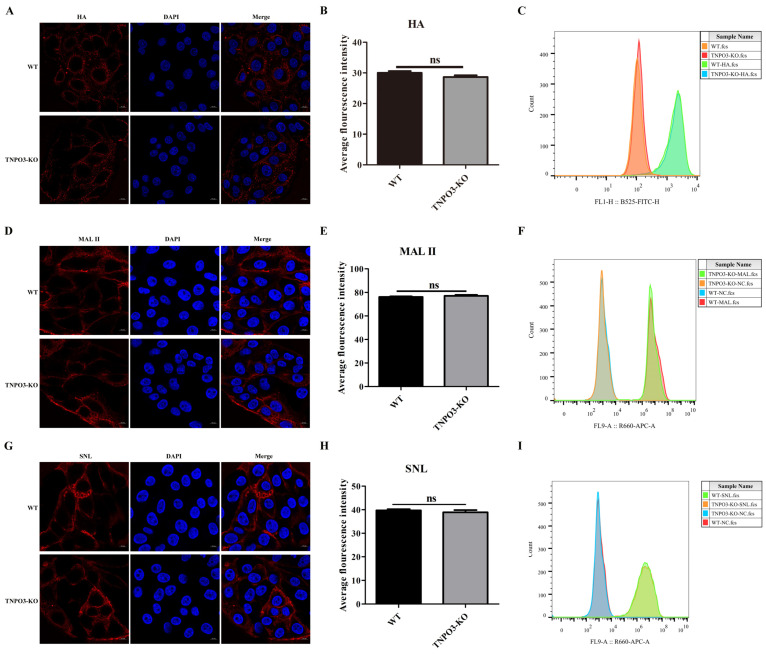
Knockout of TNPO3 does not affect viral attachment and the expression of sialic acid receptors. (**A**–**C**) The efficacy of TNPO3 KO in blocking viral attachment was assessed by an attachment assay. WT and TNPO3-KO cells were infected with HuB strain virus and incubated on ice for 1 h, followed by incubation with anti-influenza virus HA protein antibody. Then, the HA proteins were analyzed by (**A**) confocal microscopy and (**B**) analyzing the average fluorescence intensity of two independent experiments as shown in (**A**) and (**C**) flow cytometry. (**D**–**F**) Detection of α-2,3 sialic acid expression in WT and TNPO3-KO cells. WT cells and TNPO3-KO cells were incubated with α-2,3-linked sialic acid (MAL II) and then incubated with 10 µg/mL Cy5 Streptavidin and analyzed by (**D**) confocal microscopy and (**E)** analyzing the average fluorescence intensity of two independent experiments as shown in (**D**) or (**F**) flow cytometry. WT and TNPO3-KO were stained with only Cy5-streptavidin, but not treated with lectin. Scale bar = 10 μm. (**G**–**I**) Detection of α-2,6 sialic acid expression in WT and TNPO3-KO cells. WT cells and TNPO3-KO cells were incubated with α-2,6-linked sialic acid (SNL) and then incubated with 10 µg/mL Cy5 Streptavidin and analyzed by (**G**) confocal microscopy and (**H**) analyzing the average fluorescence intensity of two independent experiments as shown in (**G**) or (**I**) flow cytometry. WT and TNPO3-KO were stained with only Cy5-streptavidin, but not treated with lectin. Scale bar = 10 μm.

Although TNPO3 is involved in IAV replication, the function mechanism of TNPO3 in IAV life cycle is still unclear. We investigated the specific stages of TNPO3 knockout affecting the IAV replication. First, we tested whether the adsorption of virus particles on the host cells surface was affected by TNPO3 knockout. TNPO3-KO cells and WT cells were infected with HuB strain at MOI = 10, HA proteins were stained and analyzed by confocal microscopy and flow cytometry, and the results demonstrated that there was no difference in IAV attachment ability between TNPO3-KO cells and the WT cells (Figure 2A–C). Then, we explored whether the synthesis of the receptors was affected by TNPO3 knockout. Maackia amurensis lectin (MAL II) and Sambucus nigra lectin (SNL) can detect α-2,3 sialic acid receptor and α-2,6 sialic acid receptor on the surface of host cells, respectively [39,40]. TNPO3-KO cells and WT cells were incubated with biotin-labeled lectin and stained with Cy5-conjugated streptavidin. Confocal microscopy analyses and flow cytometry revealed that there were no differences in the synthesis of the α-2,3 sialic acid (Figure 2D–F) and α-2,6 sialic acid receptors (Figure 2G–I) between the WT cells and the TNPO3-KO cells. These data revealed that TNPO3 was not crucial for the attachment of the IAV and the expression of sialic acid receptors.

### 2.3. TNPO3 Is Not Required for the Endocytosis and Acidification

**Figure 3:**
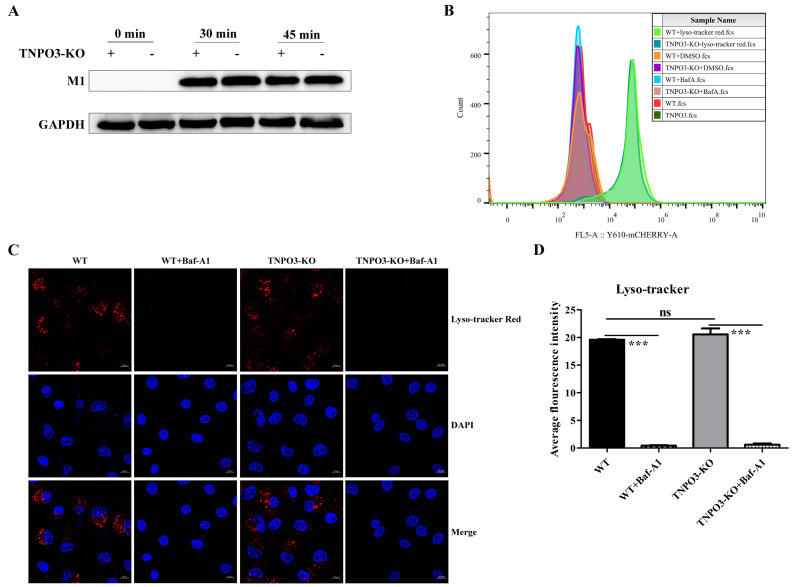
Knockout of TNPO3 does not interfere with endocytosis and acidification of IAV. (**A**) The effect of knockout of TNPO3 on viral endocytosis was assessed by Western blot analysis of viral M1 protein expression in infected WT and TNPO3-KO cells. (**B**,**C**) The effect of KO of TNPO3 on viral acidification was assessed by (**B**) flow cytometry, (**C**) confocal microscopy analysis, and (**D**) analyzing the average fluorescence intensity of two independent experiments as shown in (**C**). Baf-A1 was used as a specific inhibitor of vacuolar-type H(+)- ATPase to inhibit acidification and protein degradation in lysosomes of cultured cells. WT cells and TNPO3 KO cells were incubated either with or without Baf-A1 and then stained with Lyso-Tracker Red. Lyso-Tracker Red is a lysosome red fluorescent probe that can penetrate the cell membrane and can be used for lysosome-specific fluorescent staining of living cells. Scale bar = 10 µm. ***, *p* < 0.001; Baf-A1, Bafilomycin A1; WT, wild-type; KO, knockout.

As knockout of TNPO3 did not affect the attachment of influenza virus and the synthesis of sialic acid receptors, we continued to explore whether knockout of TNPO3 affected the subsequent endocytosis and acidification processes. For the endocytosis assay, pH 2.0 PBS was used to wash away the attached but not internalized virus particles. Internalized viral M1 protein was detected and demonstrated that the virus particles were almost undetectable at 0 min and became detectable at 30 min and 45 min post infection. However, the internalized virus particles of both the WT cells and the TNPO3-KO cells were almost the same (Figure 3A), indicating that endocytosis was not affected by TNPO3 knockout.

Next, we continued to explore the impact of TNPO3 knockout on the acidification process. Lyso-Tracker Red was utilized to detect the lysosome acidification of WT and TNPO3-KO cells. Bafilomycin A1 (Baf-A1), a known inhibitor of endosomal acidification and viral fusion, was used as a positive control [41,42]. Flow cytometry was conducted using Lyso-Tracker Red. The results indicated that the acidification process of the positive control group was completely inhibited. However, the WT cells and TNPO3-KO cells without Baf-A1 were nearly the same, revealing that the acidification step was not affected by TNPO3 depletion (Figure 3B). Additionally, Confocal microscopy was performed to observe the amount of lysosome acidification by Lyso-Tracker Red, which demonstrated that there was no difference between the WT cells and the TNPO3-KO cells (Figure 3C,D). Taking all of these results together, it was determined that TNPO3 is not required for endocytosis and acidification in the IAV replication life cycle.

### 2.4. TNPO3 Plays a Crucial Role in the Uncoating Step of Influenza Virus Entry

**Figure 4:**
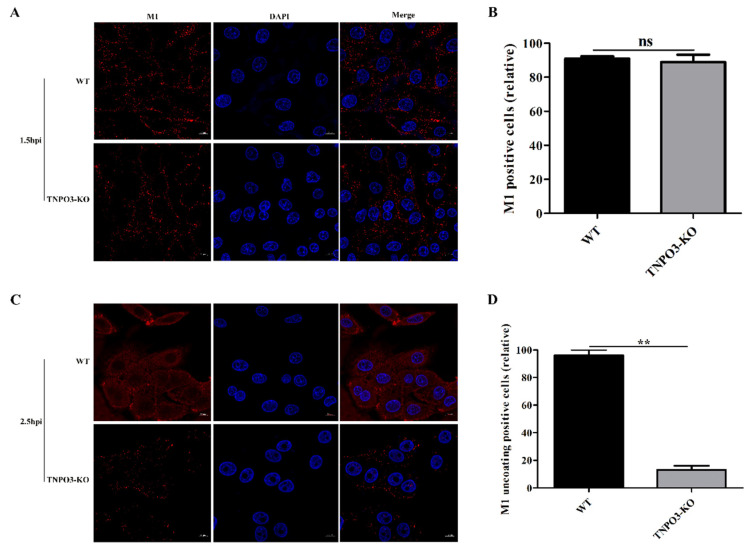
Knockout of TNPO3 inhibits uncoating during IAV entry. WT cells and TNPO3-KO cells were infected with HuB/H1N1 (MOI = 10) and fixed at (**A**,**B**) 1.5 h and (**C**,**D**) 2.5 h post infection. (**A**,**C**) Confocal microscopy analysis of viral-encoded M1 in WT and TNPO3-KO cells after IAV infection. Scale bar = 20 µm. (**B**,**D**) analyzing the relative (**B**) M1 positive or (**D**) M1 uncoating positive cells of two independent experiments as shown in (**A**) and (**C**). **, *p* < 0.01; hpi, hour post infection; WT, wild-type; KO, knockout.

Next, we investigated the effect of TNPO3 knockout on the IAV uncoating step. M2-mediated viral acidification leads to a dissociation of vRNPs from M1, resulting in the release of M1 into the cytoplasm [43,44]. After infection with HuB strain virus at MOI = 10, M1 was stained and analyzed with confocal microscopy. WT and TNPO3-KO cells shared a similar scattered and punctate M1 distribution at 1.5 hpi (Figure 4A,B). Subsequently, M1 proteins in WT cells were released and distributed throughout the cytoplasm, whereas the M1 proteins in TNPO3-KO cells still displayed a scattered and punctate distribution at 2.5 hpi (Figure 4C,D), demonstrating that the deletion of TNPO3 delayed and impaired the IAV uncoating process.

### 2.5. Knockout of TNPO3 Inhibits the Nuclear Import of IAV

**Figure 5:**
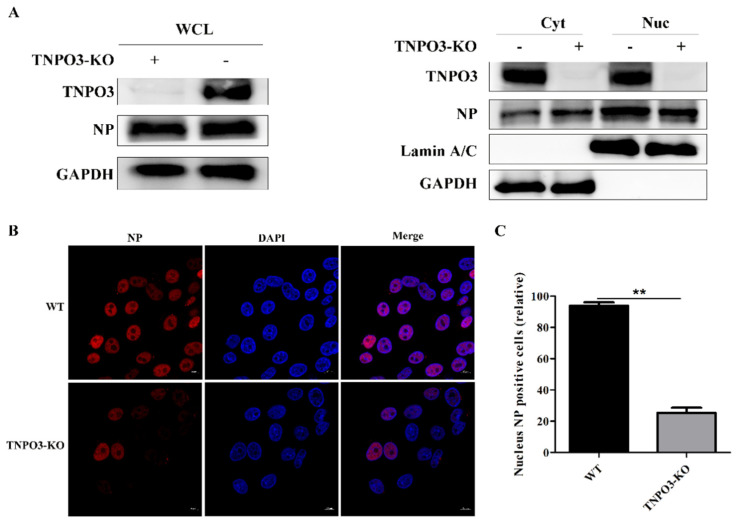
Knockout of TNPO3 inhibits IAV nuclear import. (**A**) Western blot analysis of the distribution of viral NP proteins in the cytoplasmic and nuclear fractions. WT cells and TNPO3-KO cells were infected with HuB/H1N1. Cells were harvested in 3 hpi and subjected to nuclear and cytoplasmic fractionation. Anti-NP antibody was used to determine the IAV content of the nuclear (Nuc) and cytoplasmic (Cyt) fractions as well as the whole-cell lysates (WCL). Lamin A/C was used as a nuclear protein marker and GAPDH was used as a cytosolic protein marker. (**B**) Confocal microscopy of viral-encoded NP in WT and TNPO3-KO cells after virus infection. WT and TNPO3-KO cells were infected with HuB/H1N1 and fixed at 3 hpi for observation. Scale bar = 20 µm. hpi, hour post infection; WT, wild type; KO, knockout; Nuc, nuclear; Cyt, cytoplasmic. (**C**) Analyzing the relative levels of nucleus NP positive cells in two independent experiments, as shown in B (mean ± SD of two independent experiments; **, *p* < 0.01; two-tailed Student’s *t*-test).

As the uncoating step of IAV infection was restrained by TNPO3 knockout, we explored whether the subsequent nuclear import of vRNPs into the nucleus was affected. TNPO3-KO cells and WT cells were infected with HuB strain and the viral NP proteins in nuclear and cytoplasmic fractionations were separated at 3 hpi. The results indicated that NP proteins in WT cells were distributed mostly in the nucleus, whereas much of the NP proteins were still distributed in the cytoplasm in the TNPO3-KO cells (Figure 5A), suggesting that the import of vRNPs into the nucleus was restrained. Meanwhile, confocal microscopy was also performed to visualize the distribution of the viral NP proteins at 3 hpi. NP in WT cells were observed to be primarily located in the nuclei; however, much fewer viral NP proteins were in the nucleus in the TNPO3-KO cells (Figure 5B,C). These data revealed that the inhibition of uncoating induced by TNPO3 depletion subsequently impaired the downstream import of viral vRNPs into the nucleus.

### 2.6. TNPO3 Interacts and Colocalizes with Influenza Virus M1 and M2 Proteins

**Figure 6:**
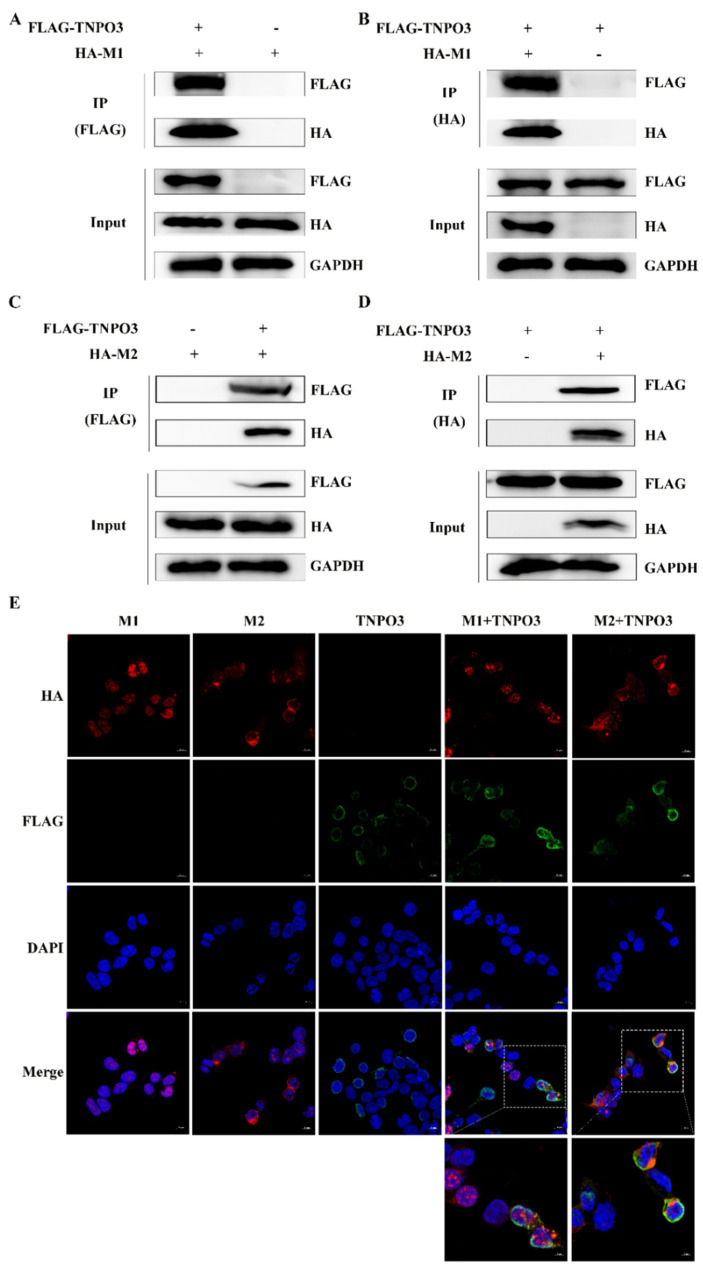
TNPO3 interacts and colocalizes with M1 and M2 proteins. (**A**,**B**) Interaction of TNPO3 with viral protein M1 detected by co-IP assay. HEK293T cells were transfected with 3 × FLAG-CMV-TNPO3 (FLAG-TNPO3) and pCAGGS-HA-M1 (HA-M1). Co-IP assays were performed with (**A**) anti-FLAG or (**B**) anti-HA antibodies followed by Western blot analysis. (**C**,**D**) Interaction of TNPO3 with viral protein M2 detected by co-IP assay. HEK293T cells were transfected with 3 × FLAG-CMV-TNPO3 (FLAG-TNPO3) and pCAGGS-HA-M2 (HA-M2). Co-IP assays were performed with (**C**) anti-FLAG or (**D**) anti-HA antibody followed by Western blot analysis. (**E**) Co-localization of TNPO3 with viral proteins M1 and M2 in HEK293T cells. HEK293T cells were transfected with 3 × FLAG-CMV-TNPO3 (FLAG-TNPO3) and pCAGGS-HA-M1 (HA-M1) or pCAGGS-HA-M2 (HA-M2), and cells were fixed at 24 hpi for observation by Confocal microscopy. “Enlarge” indicates that the picture is enlarged, scale bar = 5 µm.

We further explored the mechanism by which TNPO3 affects the uncoating of IAV. In the uncoating process of IAV, viral matrix protein M1 and M2 are mainly involved. We suspected that the TNPO3 may interact with M1 and M2 to change the structure of the virus particle protein, thus promoting the uncoating process. HEK293T cells were co-transfected with FLAG-TNPO3 and HA-M1 or HA-M2, and co-IP experiments were performed to explore the interaction between TNPO3 and M1 or M2 (Figure 6A–D). Anti-FLAG (Figure 6A) or anti-HA (Figure 6B) antibodies were utilized to precipitate interacted proteins, and the interaction between TNPO3 and M1 was validated. Meanwhile, interaction between TNPO3 and M2 was also validated by co-IP using anti-FLAG (Figure 6C) and anti-HA (Figure 6D) antibody. Moreover, confocal microscopy analyses were also conducted and validated the colocalization between TNPO3 and M1 or M2 (Figure 6E). Together, TNPO3 may interact with M1 and M2 to affect the uncoating of IAV.

## 3. Discussion

Emerging of new reassortment viruses and drug resistance to conventional vaccination and antiviral therapies raises concerns about future pandemics. Since in viruses the evolution of resistance to host-targeted therapeutics is deemed to be slow, we aimed to identify potential host factors that can act as anti-viral targets. Based on the previous genome scale screens for host factors required for IAV replication, we screened and chose TNPO3 for further research.

Uncoating is a complicated step accomplished and regulated by cellular factors to alter the virion core structure, thus resulting in the release of the viral vRNPs into the cytosol. Because of technological and methodological limitations, it is hard to observe the uncoating process and identify the host factors involved in this step. Yet, several crucial host factors have been identified to play an essential role in the uncoating process. Histone deacetylase 6 (HDAC6), G protein-coupled receptor kinase 2 (GRK2), epidermal growth factor receptor pathway substrate 8 (EPS8), and transportin-1 (TNPO1) have been validated as being involved in the influenza virus uncoating process [23,45,46,47]. Here, we identified that a new host factor, TNPO3, played a crucial role in virus uncoating through interacting with viral M1 and M2 proteins, providing new insight into the influenza virus uncoating process.

TNPO3 plays a vital role in maintaining homeostasis. The abnormal expression of TNPO3 circular RNAs (circ-RNAs) can result in ovarian cancer and gastric cancer; therefore, circ-TNPO3 has the potential to serve as a therapeutic target for cancers [48,49]. As for the antiviral targets, the import of CPSF6 by TNPO3 into the nucleus is crucial for HIV-1 replication. However, the interaction between TNPO3 and CPSF2 can be destroyed by the drug PF-3450074, inducing a significant decrease in HIV-1 virus titer [50]. Meanwhile, type I interferon (IFN)-inducible miR-128 directly targets the TNPO3 mRNA, significantly downregulating TNPO3 mRNA and the subsequent protein expression levels, thus reducing HIV-1 replication and delays the spread of infection [31]. All of this taken together, TNPO3 can serve as a potential target for antiviral therapeutics.

In our study, we confirmed the interaction and colocalization between TNPO3 and viral M1 and M2 proteins (Figure 6). As TNPO3 homologous proteins, namely TNPO1, participate in virus uncoating by binding to a PY-NLS sequence motif close to the viral M1 protein [23,51]. TNPO3 may possess a similar function to TNPO1 that binds to the specific PY-NLS-like domain of viral M1 and M2 (Figure S3) [23,52], thus changing the conformation of M1 and M2. However, the PY-NLS-like motif could not be found in the JEV and VSV viral proteins; this may be the reason why TNPO3 is not crucial for the replication of JEV and VSV. In addition, there are also possibilities that both TNPO1 and TNPO3 bind to the viral M1 protein, consequently forming a protein complex. Therefore, the binding of TNPO1 to the PY-NSL sequence may, with the help of TNPO3, TNPO1 and TNPO3, participate in the IAV uncoating process.

TNPO3 was previously found to mediate the nuclear import of splicing factor serine/arginine (SR) proteins and the serine/arginine protein CPSF6 [26,27,28,29]. Here, we described a new function of TNPO3 that assists IAV uncoating, enhancing the understanding of TNPO3. As it has nuclear importin function, TNPO3 may also function in the nuclear import of IAV vRNPs [53]. TNPO3 knockout not only affected the virus uncoating but also restrained the import of NP into the nucleus (Figure 4 and 5). Since we cannot eliminate the influence of TNPO3 knockout on restricting the virus uncoating step at present, we cannot yet further study the role of TNPO3 in vRNPs imported into the nucleus, which would provide clues for further studies related to this work.

Apart from the restriction mentioned above, there were also limitations in that IAV replication in some TNPO3-KO cells showed identical efficiency to that in WT cells. Knockout of TNPO3 may result in complex changes within the cells, including the possibility that other transportins compensated for the functions of TNPO3, as the homologous protein TNPO1 revealed similar function to TNPO3. It may be required to examine the expressions and functions of TNPO1 and TNPO2 after TNPO3 knockout. The TNPO3-KO monoclonal cells may also contain a small number of WT cells that had not be knocked out completely, resulting in a similar effect found in both the TNPO3-KO and the WT cells.

In summary, we focused on the host gene TNPO3 and explored the role of TNPO3 in IAV infection. Stepwise dissection of the viral life cycle processes revealed that TNPO3 was crucial for IAV replication and involved in the process of IAV uncoating and the subsequent importation of vRNPs into the nucleus. TNPO3 was also verified to interact and colocalize with viral M1 and M2 proteins, which may be the reason why TNPO3 affects IAV uncoating. These data establish TNPO3 as a cofactor necessary for IAV uncoating and provide a potential target for host-directed therapeutics against IAV.

## 4. Materials and Methods

### 4.1. Cell and Virus

Madin–Darby canine kidney NBL-2 (MDCK-NBL-2) cells, human embryonic kidney 293T cells (HEK293T) cells, and newborn pig tracheal epithelial cells stably expressing Cas9 protein (NPTr-Cas9) were preserved in our laboratory. The IAVs used in the experiments were A/swine/Hubei/221/2016(HuB/H1N1), A/swine/Henan/F26/2017(F26/H1N1), A/Puerto Rico/8-SV14/1934(PR8/H1N1), and A/chicken/Shanghai/SC197/2013 (SH13/H9N2). These IAVs and VSV were preserved in our laboratory. JEV P3 was kindly offered by Professor Song (Wuhan, China).

### 4.2. Antibodies and Reagents

The following antibodies and reagents were used in this study: anti-GAPDH mouse monoclonal antibody (Cat NO. 60004-1-Ig, Proteintech, Wuhan, China); anti-FLAG mouse monoclonal antibody (Cat NO. F1804, Sigma, Saint Louis, MO, USA); anti-HA mouse monoclonal antibody (Cat NO. M180-3, MBL, Beijing, China); anti-Lamin A/C rabbit polyclonal antibody (Cat NO. ET7110-12, HUABIO, Hangzhou, China); anti-TNPO3 rabbit monoclonal antibody (Cat NO. ET7108-80, HUABIO, Hangzhou, China); anti-IAV NP and M1 rabbit polyclonal antibodies (Cat NO. GTX125989 and GTX125928, GeneTex, Irvine, CA, USA); fluorescein labeled affinity purified antibody to mouse IgG (H+L) (Cat NO. 5230-0427, KPL, Milford, MA, USA) and Cy3-labeled antibody to Rabbit IgG (H+L) (Cat NO. 5230-0359, KPL, Milford, MA, USA); Horseradish peroxidase-conjugated anti-mouse and anti-rabbit antibodies (BF03001 and BF03008, Beijing Biodragon Inmmunotechnologies, Beijing, China); DAPI (4′, 6-diamidino-2-phenylindole) (Cat NO. C1002, Beyotime, Shanghai, China); biotinylated Sambucus nigra (SNL) and Maackia amurensis Lectin II (MAL II) lectins (Cat NO. B-1305-2 and B-1265-1, Vector Lab, Burlingame, CA, USA); Cy5-streptavidin (Cat NO. SA-1500-1, Vector Lab, Burlingame, CA, USA); anti-Flag Magnetic beads (Cat NO. HY-K0207, MCE, Shanghai, China); and anti-HA Magnetic beads (Cat NO. B26202, Bimake, Shanghai, China).

### 4.3. Plasmids

The sus scrofa transportin-3 (TNPO3) gene was cloned into the p3 × FLAG-CMV-14 vector at *Cla* I and *Bam* HI sites. The HuB-M1 and HuB-M2 genes were cloned into pCAGGS-HA vector at *Eco* RI and *Xho* I sites. The PCR primers were designed by Primer 5. SgRNAs were designed according to the website http://crispr.mit.edu. The primer sequences and sgRNAs are listed in Table 1. All plasmid constructs were confirmed by sequencing.

### 4.4. Generation of the TNPO3 NPTr-Cas9 Knockout Cells

TNPO3 NPTr-Cas9 knockout cells were generated by using the CRISPR/Cas9 system. Briefly, the designed single guide RNA (sgRNA) targeting the swine TNPO3 gene was cloned into the lenti-guide-puro vector. pMD2. G and psPAX2, producing the VSV-G glycoprotein and envelope proteins of the lentivirus, respectively [54,55], combined with lenti-guide-puro-sgRNA-TNPO3 (pMD2. G:psPAX2: lenti-guide-puro-sgRNA-TNPO3 = 15:6:20) were co-transfected into HEK293T cells to produce the recombined lentivirus. NPTr-Cas9 cells were infected with lentivirus, and puromycin (2.5 μg/mL) was added to select the positive clones. The monoclonal cells were obtained with the limited dilution method. Finally, the knockout of TNPO3 was confirmed by Western blot at the protein level.

### 4.5. Virus Infection and Virus Titration

NPTr-Cas9 cells were infected with IAV (MOI = 0.01) for 1 h in a 37 °C, 5% CO_2_ cell incubator, and washed three times with PBS (Cat NO. SH30256.01, HyClone, Shanghai, China). The medium was then changed to DMEM (Cat NO. SH302403.01, HyClone, Shanghai, China) with 0.25 μg/mL TPCK. The cell supernatants were collected at different time points. Viral supernatants were serially diluted with DMEM and added to each well with eight replicates of each dilution. The 50% tissue culture infective dose (TCID_50_) was calculated at 72 hpi using the Reed–Muench method [56].

### 4.6. Sanger Sequencing

The sgRNA sequence targeting TNPO3 was found in the pig genome, and PCR primers around the sgRNA sequence were designed and used to amplify the gene fragments containing the TNPO3-targeting sgRNA. Then, the gene fragments underwent Sanger sequencing to determine the genome sequence around the sgRNA of TNPO3. The primer sequences are listed in the Table 1.

### 4.7. Cell Viability Assay

Cell viabilities were assessed using a cell counting kit-8 (CCK-8) (Cat NO. GK10001, GLPBIO, Montclair, CA, USA). Assays were performed according to the manufacturer’s instructions. Briefly, the WT cells and the TNPO3-KO cells were incubated in 96-well plates and the cell viabilities were measured at 12 h, 24 h, and 36 h. A total of 10 μL of CCK-8 reagents were added to each well of the plates, and the cells were incubated at 37 °C for 1 h, then the absorbance at 450 nm was measured by a microplate reader.

### 4.8. Indirect Immunofluorescence Assay

The WT or TNPO3-KO cells were transfected with indicated plasmids or infected with HuB/H1N1. Then, cells were fixed with 4% paraformaldehyde (PFA) for 10 min, permeabilized using 0.2% (vol/vol) Triton X-100 for 10 min and blocked with 1% (wt/vol) bovine serum albumin (BSA) for 1 h. Subsequently, the cells were incubated with primary antibodies for 2 h, washed with PBS three times, and incubated with an appropriate fluorescent secondary antibody for 1 h. The nuclei were stained with DAPI for 10 min at room temperature. These samples were observed with a confocal microscope (LSM 880; Zeiss, Oberkochen, Germany).

### 4.9. Lectin and HA Binding Assay

The WT and TNPO3-KO cells were fixed with 4% PFA for 10 min, washed with PBS twice, and incubated with 20 µg/mL biotinylated lectin for 1 h on ice, followed by staining with 1 µg/mL Cy5-streptavidin. The samples were observed with a confocal microscope (LSM 880; Zeiss, Oberkochen, Germany) or analyzed by a flow cytometer (Cytoflex LX; Elementar, South Manchester, UK). As for the HA binding assay, the WT cells and the TNPO3-KO cells were infected with IAV for 1 h on ice, which allowed attachment but prevented internalization. Then, cells were fixed, permeabilized using 0.2% (*v*/*v*) Triton X-100 for 15 min, and blocked with 1% (wt/vol) bovine serum albumin (BSA) for 30 min. After that, cells were treated with primary antibodies for 2 h, followed by treatment with an appropriate fluorescent secondary antibody for 1 h. The nuclei were stained with DAPI for 10 min at room temperature. These samples were observed with a confocal microscope. [39,40]

### 4.10. Endocytosis Assay

WT cells and TNPO3-KO cells were infected with the HuB/H1N1 virus at 4 °C for 1 h, then incubated in a 37 °C cell incubator. Cells were washed with PBS (pH = 2) to remove the attached (but not the internalized) IAV at 0 min, 30 min, and 45 min. Next, the cells were collected and lysed by mammalian protein extraction reagent (Cat NO.CW0002, CWBIO, Taizhou, China). The collected proteins were subjected to a Western blot.

### 4.11. Acidification Assay

Bafilomycin A1 (Baf-A1) (Cat NO. GC17597, GLPBIO, Montclair, CA, USA) was a reagent that has been reported to inhibit the acidification process and was used as a positive control [41,42]. Lyso-Tracker Red (Cat NO. C1046, Beyotime, Shanghai, China) is an acid lysosomal dye for living cells, which can be used to indicate the pH value of acidic organelles. The cells were treated with Bafilomycin A1 (100 nM) or DMSO at 37 °C in an incubator for 2 h and then washed with PBS three times. Cells were treated with preheated Lyso-Tracker Red (100 nM) at 37 °C in an incubator under dark conditions for 1 h.

### 4.12. Uncoating Assay

WT cells and TNPO3-KO cells were infected with HuB/H1N1 at 4 °C for 1 h. After that, the cells were incubated for 1.5 h and 2.5 h at 37 °C, then fixed with 4% PFA and permeabilized with 0.25% Triton X-100. Next, cells were incubated with anti-M1 polyclonal antibody for 2 h, followed by incubating with an appropriate fluorescent secondary antibody for 1 h. The nuclei were stained with DAPI for 10 min at room temperature. Images were obtained with a confocal microscope.

### 4.13. Nuclear Import Assay

WT cells and TNPO3-KO cells were infected with HuB/H1N1 at 4 °C for 1 h, and cells were incubated at 37 °C. Cells were fixed with 4% PFA at 3 hpi. Next, cells were permeabilized with 0.25% Triton X-100, incubated with anti-NP polyclonal antibody for 2 h, and incubated with an appropriate fluorescent secondary antibody for 1 h. The nuclei were stained with DAPI for 10 min at room temperature. Images were obtained with a confocal microscope.

### 4.14. Co-IP and Western Blot Assay

HEK293T cells were transfected with the plasmids using Lipo6000 (Cat NO. 0526, Beyotime, Shanghai, China) according to the manufacturer’s instructions. After 24 h or 36 h, the cells were lysed with immunoprecipitation (IP) lysis buffer (Cat NO. P0013, Beyotime, Shanghai, China) on ice for 30 min. Subsequently, the partially lysed cells were used to conduct an immunoprecipitation assay using anti-HA magnetic beads or anti-FLAG magnetic beads. The mixture was incubated at 4 °C overnight. The immunoprecipitated proteins and remaining cell lysates were subjected to a Western blot. Images were obtained using an ECL detection system (Tanon-5200, Tanon, Shanghai, China).

### 4.15. Nuclear and Cytoplasmic Fractionation

Cells were harvested with cold PBS and resuspended in hypotonic lysis buffer (10 mM HEPES-NaOH (pH 7.9), 10 mM KCl, 1.5 mM MgCl_2_, and 0.5 mM beta-mercaptoethanol) supplemented with protease inhibitors (Cat NO. HY-K0010, MCE, Shanghai, China), vortexed, and then incubated on ice for 15 min followed by treatment with 10% NP-40 for another 5 min. Samples were centrifuged at 16,000× *g* for 10 min to obtain the cytoplasmic proteins and the pellets were used for nuclear protein extraction. The pellets, washed with cold PBS twice, were treated with mammalian protein extraction reagent (Cat NO. CW0002, CWBIO, Taizhou, China) on ice for 20 min. The samples were centrifuged at 16,000× *g* for 10 min to obtain the nuclear proteins. The proteins were used for a Western blot.

### 4.16. Statistical Analysis

Data were expressed as means ± standard deviation (SD) of three independent experiments. Statistical analysis was performed by determining *p* values using a paired two-tailed Student’s *t*-test (*, *p* < 0.05; **, *p* < 0.01; ***, *p* < 0.001, **** *p* < 0.0001).

## Figures and Tables

**Table 1:** Primers used in this study.

Primer Name	Sequence (5′ to 3′)
TNPO3-sgRNA-F TNPO3-sgRNA-R HA-M1-F HA-M1-R HA-M2-F HA-M2-R FLAG-TNPO3-F FLAG-TNPO3-R TNPO3-Genome-F TNPO3-Genome-R	CACCGTACCACGACCCAGATCCCAG AAACCTGGGATCTGGGTCGTGGTAC CGGAATTCATGAGTCTTCTAACCGAGGT CCGCTCGAGCTTGAATCGCTGTATCTGCACT CGGAATTCATGAGTCTTCTAACCGAGGT CCGCTCGAGCTCCAGCTCTATGTTGACAAAAT CCATCGATAATGAAAATGAAGATTCAGACCT CGCGGATCCTCGAAACAATCTGGTGAAGTCT AACTTCCACGGGGAACCCCT AAGCGGCGTAGATGAAGCTT

## Data Availability

Data are contained within the article.

## Supplementary Materials

The following supporting information can be downloaded at: https://www.mdpi.com/article/10.3390/ijms23084128/s1.
