# Correction: Zou et al. Transportin-3 Facilitates Uncoating of Influenza A Virus. *Int. J. Mol. Sci.* 2022, *23*, 4128

**DOI:** 10.3390/ijms262311639

**Published:** 2025-12-01

**Authors:** Jiahui Zou, Luyao Yu, Yinxing Zhu, Shuaike Yang, Jiachang Zhao, Yaxin Zhao, Meijun Jiang, Shengsong Xie, Hailong Liu, Changzhi Zhao, Hongbo Zhou

**Affiliations:** 1 State Key Laboratory of Agricultural Microbiology, College of Veterinary Medicine, Huazhong Agricultural University, Wuhan 430070, China; zoujiahui@webmail.hzau.edu.cn (J.Z.); luyaoyu0408@163.com (L.Y.); yingzizhu@163.com (Y.Z.); yangshuaike0910@163.com (S.Y.); jiachangzhao1223@163.com (J.Z.); YaxinZhao@webmail.hzau.edu.cn (Y.Z.); jiangmeijun1996@163.com (M.J.); 2 Key Laboratory of Preventive Veterinary Medicine in Hubei Province, The Cooperative Innovation Center for Sustainable Pig Production, Wuhan 430070, China; 3 Key Laboratory of Agricultural Animal Genetics, Breeding and Reproduction of Ministry of Education & Key Lab of Swine Genetics and Breeding of Ministry of Agriculture and Rural Affairs, Huazhong Agricultural University, Wuhan 430070, China; ssxie@mail.hzau.edu.cn (S.X.); hailongliu@webmail.hzau.edu.cn (H.L.); czzhao@webmail.hzau.edu.cn (C.Z.)

**Error in Figure**

In the original publication [1], there was a mistake in Figure 1 as published. In Figure 1D, the connecting line representing WT and TNPO3-KO was inverted and has been corrected accordingly; In Figure 1H, the blot labeled “Flag” should have been labeled “TNPO3.” Accordingly, the blot image has been replaced with the correct TNPO3 blot image. The corrected Figure 1 appears below. The authors state that the scientific conclusions are unaffected. This correction was approved by the Academic Editor. The original publication has also been updated.

## Figures and Tables

**Figure 1:**
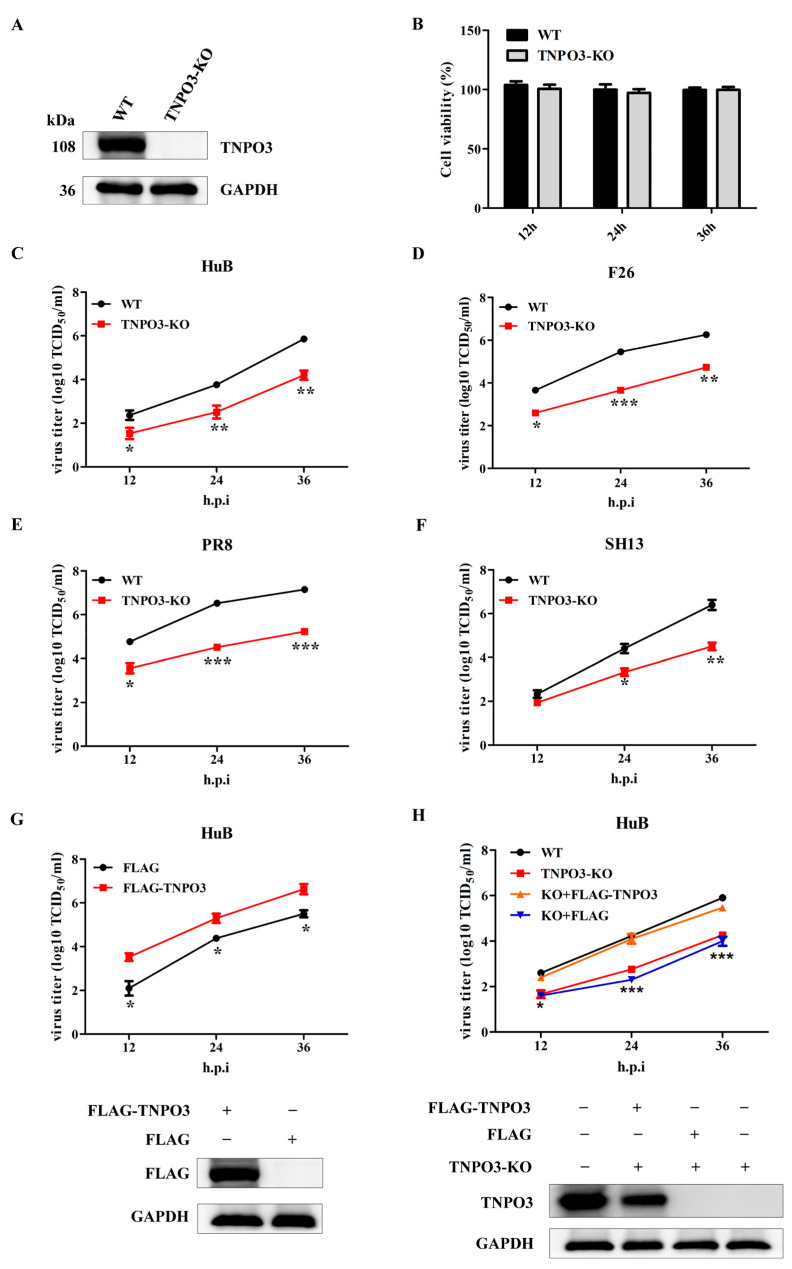
TNPO3 is involved in the replication of IAV. (**A**) Western blot detection of the TNPO3 in knockout (KO) and wild-type (WT) cells. (**B**) Cell viability was determined using CCK-8 detection. (**C**–**F**) Assessing the effects of TNPO3 knockout on IAV replication with the TCID_50_ assay. WT and TNPO3-KO cells were infected with HuB/H1N1, F26/H1N1, PR8/H1N1, and SH13/H9N2 at MOI of 0.01, respectively. (**G**) Assessment of the effect of overexpression of TNPO3 on HuB/H1N1 virus (MOI = 0.01) replication by the TCID_50_ assay. (**H**) TNPO3 knockout cells were complemented with wild-type TNPO3, and then challenged with HuB/H1N1 virus (MOI = 0.01). FLAG-TNPO3—overexpression vector of 3 × FLAG-CMV-TNPO3. FLAG—control vector of FLAG. Virus titers were determined by TCID_50_ assay on MDCK-NBL-2 cells (mean ± SD of three independent experiments; *, *p* < 0.05; **, *p* < 0.01; ***, *p* < 0.001; two-tailed Student’s *t*-test).
