# Supplementary material for: Transportin-3 Facilitates Uncoating of Influenza A Virus
Source: Int J Mol Sci. 2022 Apr 8;23(8):4128. doi: 10.3390/ijms23084128 (PMC9027869; doi:10.3390/ijms23084128)
Supplement: Supplementary file 1 [file ijms-23-04128-s001.zip › ijms-1646297-supplementary.pdf]

	PAM	
WT	CGCTCTACCACGACCCAGATCCCAGCGGAAAGG	
TNPO3-KO	CGCTCTACCACGA--------------CCCAGCGGAAAGG	-7bp

Figure S1. Sanger sequencing of TNPO3-KO and WT cells. Gene fragments contained the sgRNA aimed at TNPO3 were amplified and proceeded to sanger sequencing. An example target region with CRISPR target sites highlighted in red, and the PAM highlighted in blue.

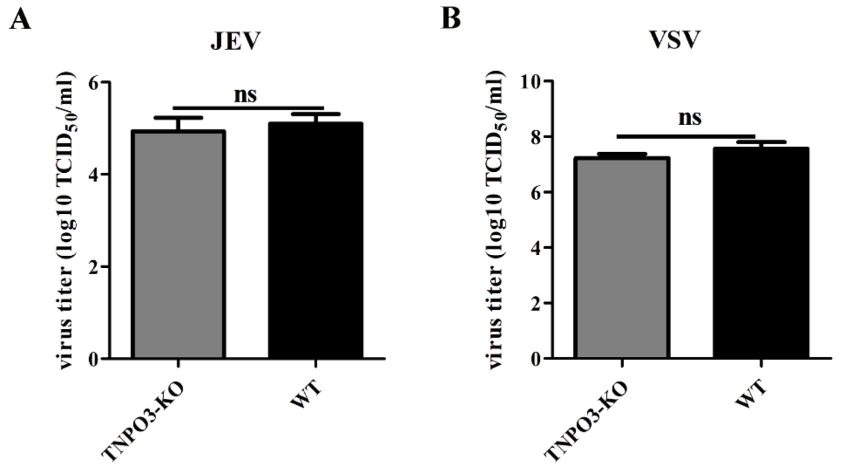

Figure S2. TNPO3 was not required for JEV and VSV replication. (A) TNPO3-KO and WT cells were challenged with JEV at MOI=0.1, and virus titers at 24 hpi were determined by $TCID_{50}$. (B) TNPO3-KO and WT cells were challenged with VSV at MOI=0.01, and virus titers at 24 hpi were determined by $TCID_{50}$. (mean ± SD of three independent experiments; *P < 0.05; **P < 0.01; ***P < 0.001; two-tailed Student's t test). hpi, hour post infection; WT, wild-type; KO, knockout.

		Epitope 1 Epitope 2 Epitope 3	
hnRNP A1	272	NFGPMKGGNFGG----R-----SSGPY	289
hnRNP D	329	GYGKV- SRRGGHQ------NSYKPY	355
FUS	507	GPGKMDSRGEHRQDRR----ERPY	526
Histone H3	11	TGGKAPRKQLATKAARK	27
IAV-M1	16	PSGPLK----------AEI AQRL	28
IAV-M2	32	I I GI LHL------ILWI LDRL	46
		Hydrophobic Basic	

Figure S3. Sequence alignment of known PY-NLSs with the conserved IAV M1 and M2 sequences. Sequences for epitopes 1 (a hydrophobic patch) highlighted in light pink, 2 (a basic patch) highlighted in Blue and 3 (PY) highlighted in Gray.
